# High Ion‐Conducting Solid‐State Composite Electrolytes with Carbon Quantum Dot Nanofillers

**DOI:** 10.1002/advs.201700996

**Published:** 2018-03-01

**Authors:** Cheng Ma, Kuan Dai, Hongshuai Hou, Xiaobo Ji, Libao Chen, Douglas G. Ivey, Weifeng Wei

**Affiliations:** ^1^ State Key Laboratory of Powder Metallurgy Central South University Changsha Hunan 410083 P. R. China; ^2^ College of Chemistry and Chemical Engineering Central South University Changsha Hunan 410083 P. R. China; ^3^ Department of Chemical and Materials Engineering University of Alberta Edmonton Alberta T6G 1H9 Canada

**Keywords:** carbon quantum dots, lithium and sodium ion batteries, nanocomposite polymer electrolytes, oxygen‐containing functional groups, poly(ethylene oxide)

## Abstract

Solid‐state polymer electrolytes (SPEs) with high ionic conductivity are desirable for next generation lithium‐ and sodium‐ion batteries with enhanced safety and energy density. Nanoscale fillers such as alumina, silica, and titania nanoparticles are known to improve the ionic conduction of SPEs and the conductivity enhancement is more favorable for nanofillers with a smaller size. However, aggregation of nanoscale fillers in SPEs limits particle size reduction and, in turn, hinders ionic conductivity improvement. Here, a novel poly(ethylene oxide) (PEO)‐based nanocomposite polymer electrolyte (NPE) is exploited with carbon quantum dots (CQDs) that are enriched with oxygen‐containing functional groups. Well‐dispersed, 2.0–3.0 nm diameter CQDs offer numerous Lewis acid sites that effectively increase the dissociation degree of lithium and sodium salts, adsorption of anions, and the amorphicity of the PEO matrix. Thus, the PEO/CQDs‐Li electrolyte exhibits an exceptionally high ionic conductivity of 1.39 × 10^−4^ S cm^−1^ and a high lithium transference number of 0.48. In addition, the PEO/CQDs‐Na electrolyte has ionic conductivity and sodium ion transference number values of 7.17 × 10^−5^ S cm^−1^ and 0.42, respectively. It is further showed that all solid‐state lithium/sodium rechargeable batteries assembled with PEO/CQDs NPEs display excellent rate performance and cycling stability.

## 1 Introduction

Solid polymer electrolytes (SPEs) for all solid‐state lithium/sodium‐ion rechargeable battery applications have been extensively investigated to satisfy several special requirements for new generation energy storage devices.^1^, ^2^ Compared with a traditional liquid electrolyte system that has safety risks caused by leakage of flammable and volatile organic solvents, SPEs possess some distinct advantages.^3^ In addition to improved safety, SPEs have excellent thermal and electrochemical stability which can extend their operating conditions to higher temperatures and higher working voltages. In addition, their flexibility allows for assembly of batteries in various package styles and their mechanical strength enables SPEs to mitigate volumetric expansion of active electrode materials and block the growth of Li dendrites.^4^, ^5^ Hence, innovative and optimized SPEs are becoming increasingly attractive, but they face several challenges.

As a typical representative of SPEs, poly(ethylene oxide) (PEO)‐based SPE is the most widely investigated system because of its high dielectric constant and strong Li^+^ solvating ability. Unfortunately, its intrinsically low ionic conductivity (10^−7^–10^−6^ S cm^−1^) originating from sluggish polymer chain dynamics upon crystallization restricts practical application.^6^ A variety of strategies, including the introduction of liquid plasticizers to produce a gel polymer electrolyte,^7^ the formation of block copolymers,^8^ crosslinking polymers^9^ and the addition of ceramic fillers^2^, ^10^ have been employed to increase the ionic conductivity of polymer electrolytes. Among these techniques, incorporating nanoscale fillers into the polymer matrix is attractive due to significant enhancement of the ion transport efficiency without sacrificing mechanical strength and electrochemical and thermal stability. It was revealed that the ionic conductivity could be raised to 10^−5^–10^−4^ S cm^−1^ at room temperature through the addition of nanoscale fillers to PEO. For instance, a PEO‐monodispersed ultrafine SiO_2_ (12 nm diameter) composite polymer electrolyte, fabricated via in situ synthesis, was reported with enhanced surface area for efficient Lewis acid‐based interaction leading to a good conductivity of 4.4 × 10^−5^ S cm^−1^ at 30 °C.^11^ Composite electrolytes consisting of Li garnet nanoparticles (≈40 nm) and a PEO matrix exhibit a high ionic conductivity of more than 10^−4^ S cm^−1^ at 30 °C.^12^ These research works also indicate that size reduction of the nanoscale fillers to tens of nanometers has a positive impact on the ionic conductivity of SPEs. However, severe particle agglomeration associated with the smaller particles makes further improvement in ionic conductivity a challenge.

With these considerations, herein we introduce a novel carbon nanostructure, i.e., carbon quantum dots (CQDs) with diameters in the 2.0–3.0 nm range, into the PEO matrix to form nanocomposite polymer electrolytes (NPEs). CQDs with high productivity and good dispersion were achieved through a simple aldol condensation reaction as reported in our previous study,^13^ demonstrating rich oxygen‐containing functional groups on the CQD surfaces. PEO/CQDs NPEs were obtained by blending CQDs, PEO, and LiClO_4_ or NaClO_4_ salts. The effects of CQDs on ion transport behavior and physicochemical properties of PEO/CQDs NPEs are investigated. We show that PEO/CQDs NPEs exhibit excellent ionic conductivities and high lithium/sodium ion transference numbers, which originate from highly dispersed CQDs with oxygen functional groups that can effectively promote dissociation of LiClO_4_ or NaClO_4_ salts and adsorption of ClO_4_^−^ anions, and increase the amorphicity of PEO matrix. Importantly, with these PEO/CQDs NPEs, assembled Li/LiFePO_4_ (LFP) and Na/Na_3_V_2_(PO_4_)_3_ (NVP) rechargeable batteries display improved long‐term cyclability and rate performance.

## 2 Results and Discussion

### 2.1 Characterization of As‐Prepared CQDs

**Figure 1:**
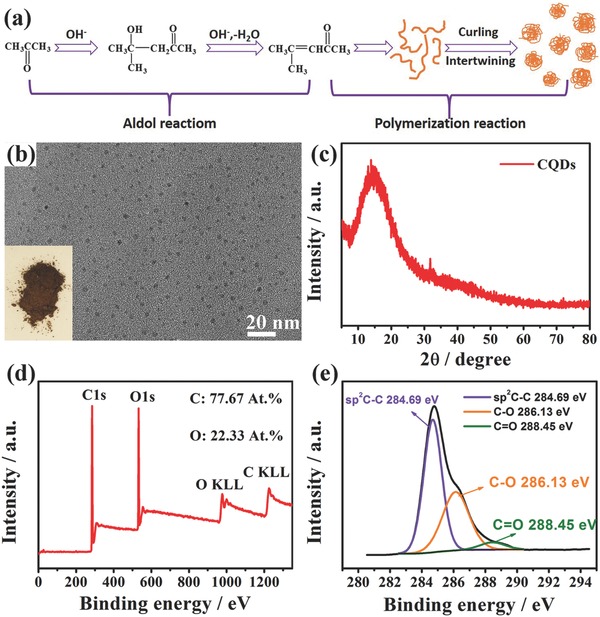
Synthesis and characterization of as‐prepared CQDs. a) Schematic illustration of the synthesis mechanism for CQDs. b) TEM bright field (BF) image and optical image (inset). c) XRD pattern. d) XPS survey spectrum. e) High‐resolution C 1s XPS spectrum.

A schematic illustration of the synthesis mechanism for CQDs is presented in **Figure** **1**a. The aldol reaction of acetone first occurs in the presence of NaOH and leads to the formation of an unsaturated ketone.^14^ In the follow‐up process, the polymerization reaction of the unsaturated ketone results in oligomer products with extended carbon chains and these oligomer chains eventually produce CQDs through curling and intertwining induced by thermal motion.^13^ As shown in Figure 1b, the obtained CQDs are brown powders and a typical transmission electron microscopy (TEM) image demonstrates that the CQDs are well dispersed with an average diameter of 2.0–3.0 nm. In addition, CQDs have excellent solubility in different organic solvents without ultrasonic treatment (Figure S1, Supporting Information). An X‐ray diffraction (XRD) pattern of the CQDs is presented in Figure 1c, and only one broad peak centered at 16° is visible, indicative of an amorphous phase.

On the basis of ^1^H NMR and mass spectra (Figure S2, Supporting Information), the prepared CQDs are confirmed to be a mixture of oligomers with molecular weights in the range of 400–1000 g mol^−1^. X‐ray photoelectron spectroscopy (XPS) measurements were utilized to clarify the elemental composition and carbon bonding configurations of the CQDs (Figure 1d). The CQDs mainly contain carbon (77.7 at%) and oxygen (22.3 at%). The high‐resolution C1s spectrum (Figure 1e) can be deconvoluted into three peaks at binding energies of 284.69, 286.13, and 288.46 eV corresponding to sp^2^ carbon atoms, alcoholic (C—O) species and carbonyl (C=O) species, respectively.^15^ Oxygen‐rich functional groups are also evident in the Fourier transformation infrared (FTIR) spectrum and UV–vis absorption spectrum (Figure S3, Supporting Information). In addition, Figure S4 in the Supporting Information depicts thermogravimetric analysis of the CQDs, demonstrating that absorbed moisture as well as the low molecular weight components evaporated as the temperature was increased; noticeable weight loss started at ≈160 °C.

### 2.2 Morphology, Mechanical, and Electrochemical Performance of PEO/CQDs NPEs

**Figure 2:**
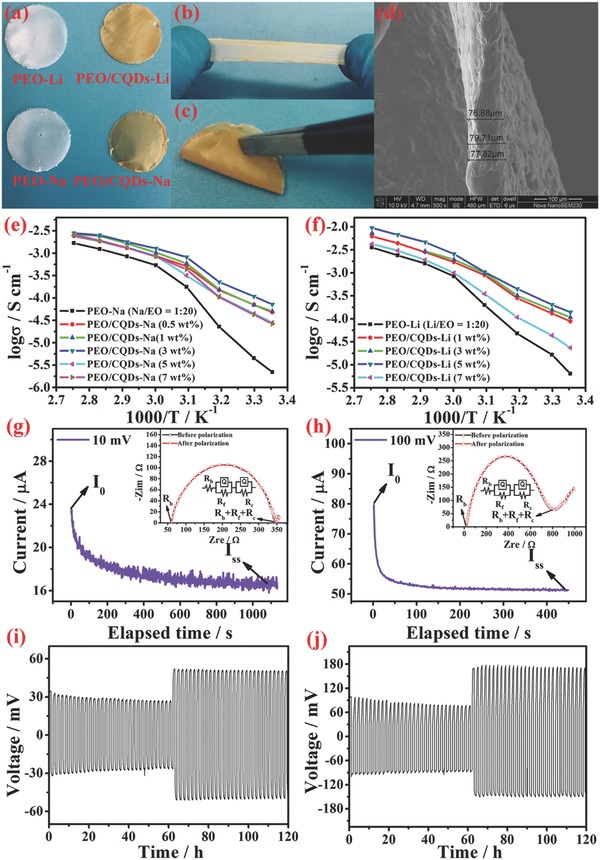
Morphology, Na/Li ion transport properties, and interfacial stability for PEO/CQDs NPEs. a) Digital photograph of different PEO polymer electrolyte membranes. b,c) Tension property and bending performance of PEO/CQDs electrolyte membranes. d) Representative cross‐sectional SEM image of PEO/CQDs electrolyte membrane. Temperature‐dependent ionic conductivities for e) PEO/CQDs‐Na and f) PEO/CQDs‐Li with various CQD contents. Chronoamperometry profiles and AC impedance spectra before and after polarization for g) symmetric Li/Li cells with PEO/CQDs‐Li and h) symmetric Na/Na cells with PEO/CQDs‐Na. Voltage profiles for lithium/sodium plating/striping tests taken from i) PEO/CQDs‐Li membrane in a symmetric Li/Li coin cell and j) PEO/CQDs‐Na membrane in a symmetric Na/Na coin cell at current densities of 0.05 and 0.1 mA cm^−2^ at 60 °C.

**Figure** **2**a shows that the PEO membranes are semitransparent and freestanding, while the color changes from white (PEO SPEs) to brownish yellow (PEO/CQDs NPEs). Tensile and bending tests (Figure 2b,c) indicate that the PEO/CQDs NPEs exhibit excellent mechanical flexibility and the membrane thickness is estimated to be about 80 µm (Figure 2d). To understand the effect of CQDs on the mechanical properties of PEO matrix, dynamic mechanical analysis (DMA) was carried out with a sweeping frequency of 0.01–100 Hz. The stress–strain curves for the composite electrolyte membranes are shown in Figure S5 in the Supporting Information. It is noted that the introduction of CQDs leads to reduction in mechanical strength and improvement in ductility in both Li and Na salt‐containing PEO electrolytes, which may arise from the plasticization of PEO matrix by CQDs. A similar trend was also observed in previous studies on succinonitrile‐ and metal‐organic frameworks (MOF)‐modified PEO composites.^16^

Ionic conductivity is a key parameter for the application of polymer electrolytes in high‐performance rechargeable batteries.^17^ The conductivities of PEO‐Na electrolytes with different concentrations of NaClO_4_ salt were investigated and the maximum conductivity value was obtained for the sample with Na/ethylene oxide (EO) = 1/20, i.e., 10.79 wt% of NaClO_4_ salt (Figure S6, Supporting Information). For convenient comparison, therefore, Na/EO and Li/EO ratios of 1/20 were selected as the doped concentration of NaClO_4_ and LiClO_4_ salts in NPEs. Figure 2e,f presents Arrhenius plots of ionic conductivity with temperatures ranging from 25 to 90 °C for PEO/CQDs NPEs. When compared with the ionic conductivity of bare PEO SPEs, substantial enhancements are observed for PEO/CQDs‐Na NPE and PEO/CQDs‐Li NPE due to the homogeneous dispersion of CQDs within the PEO matrix. At ambient temperature, the highest ionic conductivities for PEO/CQDs‐Na NPE and PEO/CQDs‐Li NPE are 7.17 × 10^−5^ and 1.39 × 10^−4^ S cm^−1^, respectively (Table S1, Supporting Information), and the values are also contrasted with other nanofiller doped PEO polymer electrolytes (Table S2, Supporting Information).^11^, ^18^, ^19^ As for PEO/CQDs‐Na NPE, the optimal CQD content corresponding to the maximum conductivity value is 3 wt%, which is lower than that for PEO/CQDs‐Li NPE (5 wt%). This difference may be related to the ionic radius difference between sodium ions and lithium ions.

In addition to the enhanced ionic conductivities, high lithium/sodium ion transference numbers (*t^+^*) are also achieved for PEO/CQDs NPEs. Figure 2g,h shows the variations in current with time and the AC impedance spectra before and after polarization. According to Equation (2) in the Experimental Section, *t^+^* for the PEO/CQDs‐Li and PEO/CQDs‐Na NPE are estimated to be 0.48 and 0.42, respectively, which are superior to the values for PEO‐Li (0.21) and PEO‐Na (0.18) SPE (Figure S7, Supporting Information) and other PEO‐based SPEs.^18^, ^20^ The high ion transference numbers indicate strong interactions between oxygen functional groups of CQDs and alkali salts, which enhances the rate capability of rechargeable batteries by reducing the concentration polarization during the charge–discharge process.^17^

Polarization tests of the Li/Li and Na/Na symmetrical cells were performed under constant current densities of 0.05 and 0.1 mA cm^−2^ at 60 °C to investigate the effect of the PEO/CQDs NPEs on the lithium and sodium plating/striping processes. Figure 2i,j shows the corresponding time‐dependent voltage profiles for the Li/Li and Na/Na symmetrical cells. Generally, the polarization voltage for Na/Na cell is higher than that for Li/Li cell, which is consistent with the ionic conductivity difference observed in Figure 2e,f. Moreover, for both Li/Li and Na/Na symmetrical cells, the polarization voltage remains stable after the initial 30 h, without random variations occurring during the following cycles. The experimental results above indicate that the PEO/CQDs NPEs may promote uniform plating and striping of lithium and sodium, and in turn, effectively inhibit the growth of lithium or sodium dendrites.

Electrolyte stability, both electrochemical and thermal, is also critical for high‐energy rechargeable batteries.^5^, ^21^ As shown in Figure S8 in the Supporting Information, PEO/CQDs‐Li and PEO/CQDs‐Na NPE are stable within 4.5 V versus Li^+^/Li and Na^+^/Na, respectively, while bare PEO SPEs generally show vlower anodic oxidation potentials. It is well documented that the anodic breakdown of anions accounts for electrochemical instability at high potentials.^22^ The enhancement of electrochemical stability suggests that the CQDs exert a strong adsorption effect on the anions and, subsequently, suppress their anodic decomposition processes. In addition, the low decomposition temperature of CQDs results in no obvious deterioration of the thermal stabilities for PEO/CQDs‐Li and PEO/CQDs‐Na NPEs. These results suggest improved safety performance of PEO/CQDs NPEs compared with conventional liquid electrolytes.

### 2.3 Mechanism of Improved Electrochemical Performance for PEO/CQDs NPEs

**Figure 3:**
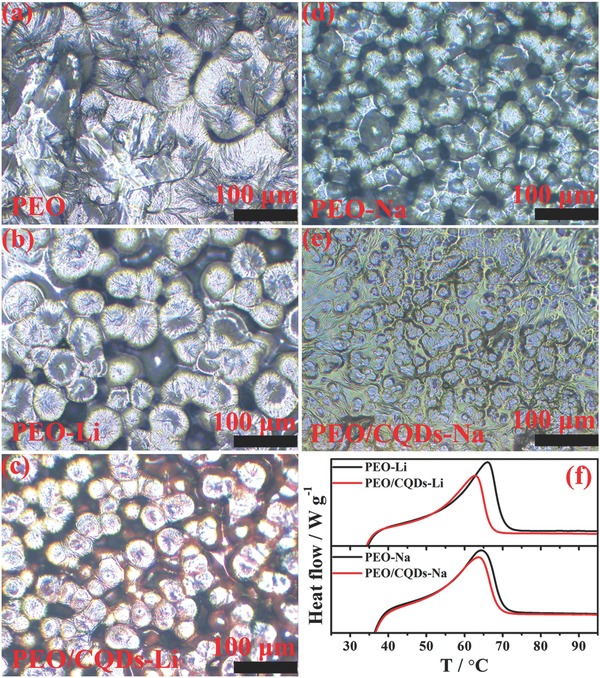
Crystallization behavior of different electrolytes. a–e) PLM images of pure PEO, PEO‐Li, PEO/CQDs‐Li, PEO‐Na, and PEO/CQDs‐Na. f) DSC curves for PEO‐Li, PEO/CQDs‐Li, PEO‐Na, and PEO/CQDs‐Na.

Enhanced Li/Na ion transport is evident in NPEs with CQDs that are enriched with oxygen‐containing functional groups. In order to understand the working mechanism associated with the improved electrochemical performance, microscopic and spectroscopic characterizations were employed to evaluate the crystallization and salt dissociation behavior in the NPEs. Very low contrast and serious radiation damage induced by electron beam make electron microscopies, such as scanning electron microscopy (SEM) and TEM, difficult to differentiate PEO matrix and CQDs effectively. On the contrary, polarized light microscopy (PLM) is an effective technique to identify samples that contain both crystalline and amorphous components at the micrometer‐scale.^23^, ^24^ **Figure** **3**a,e presents PLM images of pure PEO, PEO‐Li, PEO/CQDs‐Li, PEO‐Na, and PEO/CQDs‐Na. The PEO‐Li and PEO‐Na electrolytes contain smaller crystalline zones compared with the pure PEO polymer matrix. In addition, the crystal size is further reduced with the addition of CQDs and more amorphous regions exist in the PEO/CQDs NPEs. Note that the PLM images provide barely qualitative information on crystalline and amorphous phases of the PEO matrix. In order to quantify the fraction of crystalline component, differential scanning calorimetry (DSC) analysis was carried out on NPEs with various amounts of CQDs, as shown in Figure S9 and Tables S3 and S4 in the Supporting Information. Specifically, DSC curves taken from PEO/CQDs (5 wt%)‐Li and PEO/CQDs(3 wt%)‐Na are compared in Figure 3f to show the change in crystallinity. With the addition of CQDs, lower melting temperatures (*T*_m_) and melting enthalpies (Δ*H*_m_) are detectable, which is indicative of lower degrees of crystallinity (χ_c_) for PEO/CQDs‐Li NPE and PEO/CQDs‐Na NPE, respectively (see detailed information in Tables S3 and S4, Supporting Information). A possible reason is that CQDs with favorably chemical compatibility effectively disrupt the regularity of PEO segments.

**Figure 4:**
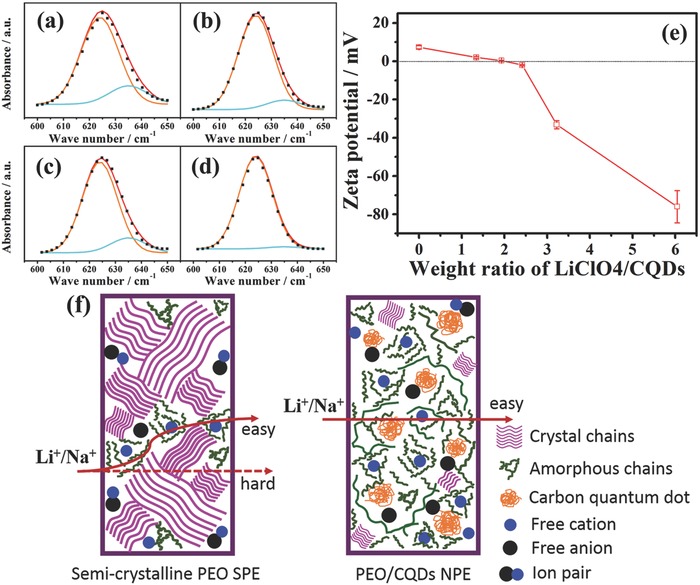
Characterization of the interaction behavior between CQDs and alkali salts. a–d) FTIR spectra at 600–650 cm^−1^ and corresponding Gaussian–Lorentzian fitting curves for a) PEO‐Li, b) PEO/CQDs‐Li, c) PEO‐Na, and d) PEO/CQDs‐Na. e) Zeta potential of LiClO_4_/CQDs samples with different weight ratios in alcohol suspension. f) Schematic illustration showing ion transport mechanism in PEO SPE and PEO/CQDs NPE.

The dissociation of alkaline salts in PEO‐based electrolytes is also crucial for enhanced ionic conductivity. FTIR analysis has been widely used to estimate the fraction of free anions.^11^, ^25^ As shown in **Figure** **4**a–d, FTIR spectra with wave numbers in the range of 600–650 cm^−1^ can be deconvoluted into two separate bands: one band centered at 624 cm^−1^ is assigned to the free anions and the peak at 635 cm^−1^ is associated with ion pairs. As summarized in Table S5 in the Supporting Information, the dissociation ratios for PEO/CQDs‐Li NPE and the PEO/CQDs‐Na NPE increase to 92.7% and 97.9%, respectively, which are significantly higher than those for PEO‐Li SPE (82.5%) and PEO‐Na SPE (86.1%). These results suggest that oxygen functional groups on the surface of CQDs can interact strongly with LiClO_4_/NaClO_4_ salts and then release more free ions. The interaction between CQDs and alkali salts is also confirmed by zeta potential measurements. Figure 4e shows the zeta potential of LiClO_4_/CQDs samples with different weight ratios. As the ratio increases, the zeta potential decreases from positive to negative. The zeta potential is around zero at a weight ratio of 1.93 corresponding to the highest ionic conductivity in PEO/CQDs‐Li NPE, which reveals adsorption of ClO_4_^−^ anions at the surface of CQDs by positively charged Lewis acid sites.^23^, ^26^

Taking the above information into account, a working mechanism on the enhanced Li^+^/Na^+^ ion transport efficiency in the PEO/CQDs NPEs is depicted schematically in Figure 4f. For semicrystalline PEO SPE, there exist a limited number of noncontinuous amorphous chains that can dissociate alkaline salts and transfer Li^+^/Na^+^; the long and flexural ion transmission path results in low ionic conductivity.^27^ In contrast, the addition of well‐dispersed CQDs results in strong Lewis acid–base interactions among CQD, LiClO_4_/NaClO_4_, and PEO. These interactions not only increase the free volume and segmental motion by reducing the crystallinity of PEO polymer matrix but also enhance the dissociation of Li/Na salts and adsorption of ClO_4_^−^ anions, all of which facilitate Li^+^/Na^+^ ion transport through the creation of low‐energy ion conduction pathways along the CQD and polymer matrix interface.

### 2.4 All‐Solid‐State Li/Na Battery Performance

**Figure 5:**
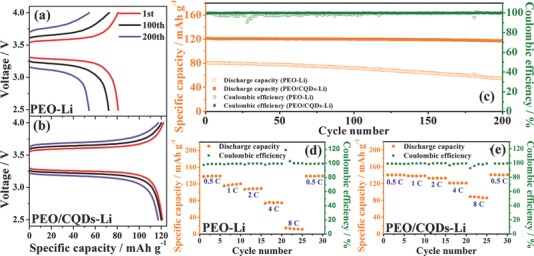
All‐solid‐state LFP/Li battery performance. a,b) Charge/discharge curves at different cycles of LFP/Li batteries using PEO‐Li and PEO/CQDs‐Li electrolytes at 4 C. c) Capacity and Coulombic efficiency versus cycle number for batteries using PEO‐Li and PEO/CQDs‐Li electrolytes at 4 C rate. d,e) Comparison of rate capacity for batteries with PEO‐Li and PEO/CQDs‐Li electrolytes. All the measurements were conducted at 60 °C.

In order to further demonstrate the improved electrochemical performance of PEO/CQDs‐Li NPE, cycling stability and rate capacity for LFP/Li battery are compared in **Figure** **5**. The charge and discharge profiles at different cycle numbers at 4 C and 60 °C, as shown in Figure 5a,b, indicate that the overpotential of the PEO/CQDs‐Li NPE‐based battery is smaller than that of the PEO‐Li‐based battery, demonstrating lower polarization and enhanced cycling stability for the LFP/Li battery with PEO/CQDs‐Li NPE. The LFP/Li battery with PEO/CQDs‐Li NPE can deliver an initial discharge capacity of 121 mAh g^−1^ with a capacity retention rate of 97.1% after 200 cycles at 4 C and 60 °C (Figure 5c). The coulombic efficiency approaches nearly 100% upon cycling. In contrast, the PEO‐Li‐based LFP/Li battery displays a much lower initial discharge capacity (80.7 mAh g^−1^) at 4 C and the capacity retention rate is 67.2% and a significantly larger polarization after 200 cycles, respectively (Figure 5c). Figure 5d,e presents a comparison of rate capacities at various C‐rates. The PEO/CQDs‐Li NPE‐based battery also shows a much better rate capability, the specific discharge capacities at 0.5, 1, 2, 4, and 8 C are 140, 138, 133, 121, and 88 mAh g^−1^, respectively. The corresponding capacities for the PEO‐Li‐based battery are 136, 118, 108, 75, and 13 mAh g^−1^. As seen in the discharge curves at various C‐rates with a charge current density of 0.1 C for LFP/Li batteries (Figure S10, Supporting Information), the PEO/CQDs‐Li‐based cell exhibits a more complete discharge platform and enhanced capacity retention at high C‐rates, which is mainly attributed to the higher ionic conductivity and Li^+^ ion transference number of PEO/CQDs‐Li NPE.

**Figure 6:**
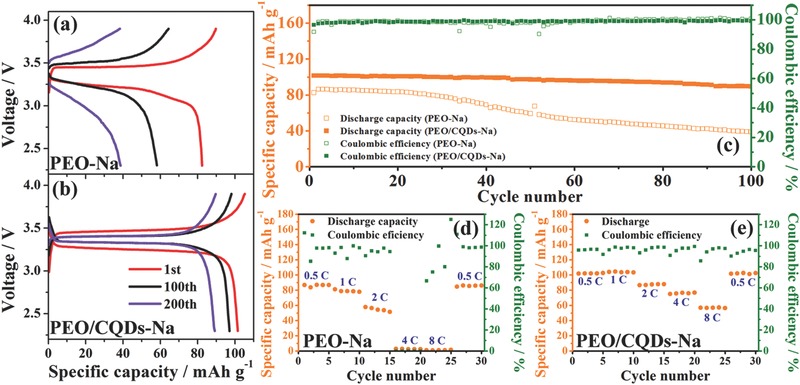
All‐solid‐state NVP/Na battery performance. a,b) Charge/discharge curves at different cycles of NVP/Na batteries using PEO‐Na and PEO/CQDs‐Na electrolytes at 1 C. c) Capacity and Coulombic efficiency versus cycle number for batteries using PEO‐Na and PEO/CQDs‐Na electrolytes at 1 C rate. d,e) Comparison of rate capacity for batteries with PEO‐Na and PEO/CQDs‐Na electrolytes. All the measurements were conducted at 60 °C.

A Na‐superionic conductor (NASICON)‐type structured Na_3_V_2_(PO_4_)_3_ electrode was chosen to evaluate the feasibility of using PEO/CQDs‐Na NPE in a sodium battery. As shown in **Figure** **6**a–c, the PEO/CQDs‐Na‐based battery gives an initial discharge capacity of 101.5 mAh g^−1^ and stabilizes at 89.4 mAh g^−1^ after 100 cycles at 1 C. The NVP/Na battery with the PEO‐Na electrolyte exhibits a lower discharge capacity of 86.32 mAh g^−1^ and an inferior capacity retention of 45.1% after 100 cycles. Rate performance (Figure 6d,e) and discharge profiles at different C‐rates (Figure S11, Supporting Information) were also compared. It is evident that substantial improvement in rate capability can be observed in the PEO/CQDs‐Na‐based battery. High discharge capacities (about 103 mAh g^−1^) are achieved at 0.5 C and 1 C, which are close to the theoretical value of 118 mAh g^−1^ for NVP. At very high rates of 4 C and 8 C, the specific capacities of the PEO/CQDs‐Na‐based battery are 76 and 56 mAh g^−1^, respectively, while no reversible capacity was detected for the NVP/Na battery with the PEO‐Na electrolyte. These results further confirm the improved electrochemical properties of PEO/CQDs‐Na NPE, which are ascribed to its high ionic conductivity and Na^+^ transference number.

## 3 Conclusions

Well‐dispersed CQDs with oxygen‐containing functional groups were fabricated through a simple and low energy consumption method. Highly dispersed CQDs and strong Lewis acid–base interactions effectively increased the dissociation degree of LiClO_4_ or NaClO_4_, adsorption of ClO_4_^−^ anions as well as the amorphous phase content of PEO. As such, nanocomposite PEO‐based polymer electrolytes of PEO/CQDs‐Li and PEO/CQDs‐Na exhibit high ionic conductivities of 1.39 × 10^−4^ and 7.17 × 10^−5^ S cm^−1^, respectively, and a high Li ion transference number of 0.48 (PEO/CQDs‐Li) and Na ion transference number of 0.42 (PEO/CQDs‐Na). Moreover, PEO/CQDs NPEs with excellent electrochemical characteristics enable the fabrication of all solid‐state Li and Na batteries with significantly enhanced cycling stability and rate performance. Both the simple fabrication process and the outstanding electrochemical performance of PEO/CQDs NPEs make them promising candidates for application in next generation solid‐state rechargeable batteries.

## 4 Experimental Section

*Preparation of CQDs*: The CQDs were prepared by a facile aldol condensation process, as reported in our previous study.^13^ Specifically, 8 g of NaOH was dissolved into 30 mL of acetone (C_3_H_6_O, analytical reagent (AR) grade) under constant stirring for 1 h, followed by natural aging at ambient temperature in air. After 96 h, the resulting solid mixture was neutralized with dilute HCl solution, separated centrifugally, and washed with deionized water. Then the final product was dried in a vacuum at 100 °C for 12 h producing a brown CQD powder.

*Preparation of PEO/CQDs NPEs*: PEO (*M*_W_ = 6 × 10^6^ g mol^−1^, Sigma‐Aldrich), lithium perchlorate (LiClO_4_, 99.99%, Aladdin), and sodium perchlorate (NaClO_4_, 99.99%, Aladdin) were dried under vacuum before use. PEO/CQDs films were prepared using a solution casting technique. PEO, CQDs, and LiClO_4_/NaClO_4_ were dissolved in acetonitrile (CH_3_CN, AR grade) and the solution was stirred for 12 h. This was followed by casting on a polytetrafluoroethylene (PTFE) plate and drying at 70 °C in a vacuum oven for 24 h. The obtained electrolyte films were then transferred into an argon‐filled glove box and stored for at least 24 h before electrochemical measurements.

*Materials Characterization*: TEM (JEOL Japanese electronics maker (JEM)‐2100F) and SEM (Nova Nano SEM 230) were utilized to characterize the morphology of the samples. Compositional and structural information of the products were investigated by powder XRD (Rigaku D/Max‐2500) with Cu *K*_α_ radiation (λ = 1.5406 Å), nuclear magnetic resonance spectroscopy (Avance III 400 MHz Digital NMR spectrometer), FTIR spectroscopy (Nicolet 6700), XPS (K‐Alpha 1063), and UV absorption spectrometry (UV‐1801). DSC (simultaneous DSC‐TGA Q series (SDTQ)‐600 TA) and thermogravimetric analysis (TG, DTA6300) measurements were performed at a heating rate of 10 °C min^−1^ under N_2_ atmosphere. Zeta potential and surface charge characteristics of the CQDs nanofillers in the LiClO_4_ ethanol solution were measured using a Zetasizer Nano ZS (Malvern Instruments, UK). The mechanical properties of the electrolyte membrane were measured using a dynamic mechanical analysis (DMA) Q800 (TA instruments) with a sweeping frequency of 0.01–100 Hz and the dimensions of the electrolyte sample are 0.6 cm × 4 cm × 80 µm.

*Electrochemical Measurements*: The ionic conductivity was calculated from the electrolyte resistance (*R*_b_), the thickness of electrolyte film (*L*), and the electrode area (*A*), according to Equation (1). The electrolyte resistance was measured using electrochemical impedance spectroscopy with symmetric SS (stainless steel)/SPE/SS cells in the frequency range of 100 KHz to 0.1 Hz with a 10 mV voltage amplitude.

(1) $$\sigma =L/R_{b}A$$

Linear sweep voltammetry was employed to determine the electrochemical stability of the polymer electrolytes in SS/SPE/Li or SS/SPE/Na coin cells at a scanning rate of 1 mV s^−1^. The Li/Na ion transference numbers were evaluated by combined DC polarization/AC impedance in symmetric Li/SPE/Li and Na/SPE/Na cells. The current values from initial (*I*_0_) to steady state (*I*_ss_) conditions under a polarization potential (Δ*V*) were recorded and the interfacial resistances (*R*_0_ and *R*_ss_) were also examined before and after polarization by AC impedance. The transference number, *t^+^*, was measured and calculated from the following equation

(2) $$t^{+}=\frac{I_{\mathrm{ss}}\left(\Delta V-I_{0}R_{0}\right)}{I_{0}\left(\Delta V-I_{\mathrm{SS}}R_{\mathrm{SS}}\right)}$$

*Battery Testing*: LFP powders (Aleees Inc.), NVP powders (prepared according to ref. 28), carbon black (Yiborui Chemical Industry Company, Tianjin, China), and poly(vinylidene fluoride) (PVDF, Arkema Inc.) were dried under vacuum before use. To prepared positive electrodes, slurries containing LFP or NVP, PVDF, and carbon black with a ratio of 75:15:10 were dispersed in *N*‐methyl‐2‐pyrrolidone (NMP, Aladdin) and the resultant slurries were coated onto aluminum foil and dried in a vacuum oven at 110 °C for 12 h to remove the residual NMP. The mass loading of the active material on the positive electrode was 2.0–2.2 mg cm^−2^. The positive electrodes and electrolyte membranes were housed and sealed in 2016 coin cells to fabricate LFP/SPE/Li and NVP/SPE/Na batteries. All coin‐type cells were assembled in an argon‐filled glove box and the charge–discharge tests were carried out using a battery testing system (LANHE CT2001A, Wuhan LAND Electronics Co.).

## Conflict of Interest

The authors declare no conflict of interest.

## Supporting information

Supplementary
