# Supplementary material for: High Ion‐Conducting Solid‐State Composite Electrolytes with Carbon Quantum Dot Nanofillers
Source: Adv Sci (Weinh). 2018 Mar 1;5(5):1700996. doi: 10.1002/advs.201700996 (PMC5980199; doi:10.1002/advs.201700996)
Supplement: Supplementary file 1 — Supplementary [file ADVS-5-1700996-s001.pdf]

Copyright WILEY-VCH Verlag GmbH & Co. KGaA, 69469 Weinheim, Germany, 2018.

ADVANCED SCIENCE Open Access

Supporting Information

for *Adv. Sci.,* DOI: 10.1002/advs.201700996

High Ion-Conducting Solid-State Composite Electrolytes with Carbon Quantum Dot Nanofillers

*Cheng Ma, Kuan Dai, Hongshuai Hou, Xiaobo Ji, Libao Chen, Douglas G. Ivey, and Weifeng Wei**

Copyright WILEY-VCH Verlag GmbH & Co. KGaA, 69469 Weinheim, Germany, 2016.

Supporting Information

High Ion-conducting Solid-State Composite Electrolytes with Carbon Quantum Dot NanoFillers

Cheng Ma [a], *Kuan Dai* [a], *Hongshuai Hou* [a, b], *Xiaobo Ji* [b], *Libao Chen* [a], *Douglas G. Ivey* [c] *and Weifeng Wei* [a,] *

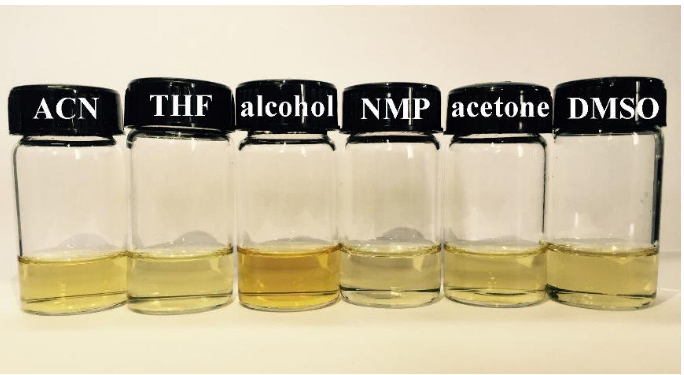

Figure S1. Digital photographs of carbon quantum dots (CQDs) dissolved in various organic solvents.

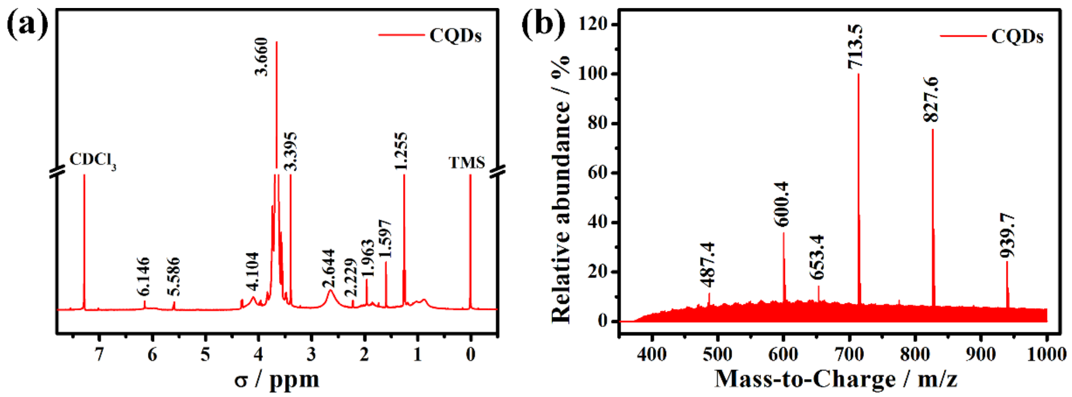

Figure S2. (a) ^{1}H NMR and (b) mass spectra of CQDs.

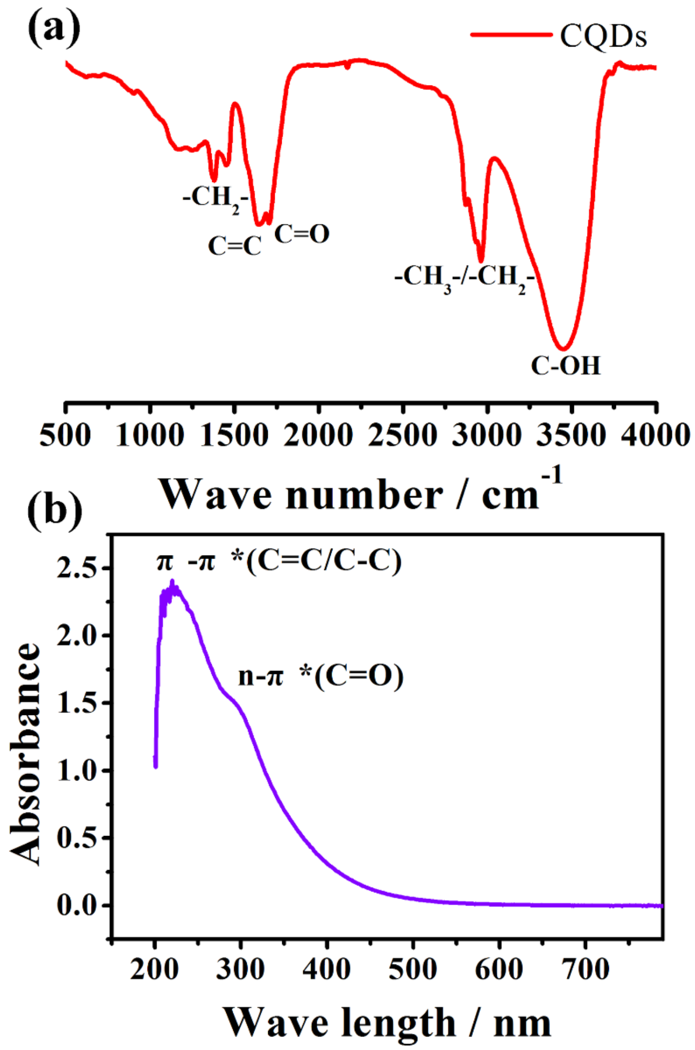

Figure S3. (a) FTIR spectrum and (b) UV-vis absorption spectrum of CQDs.

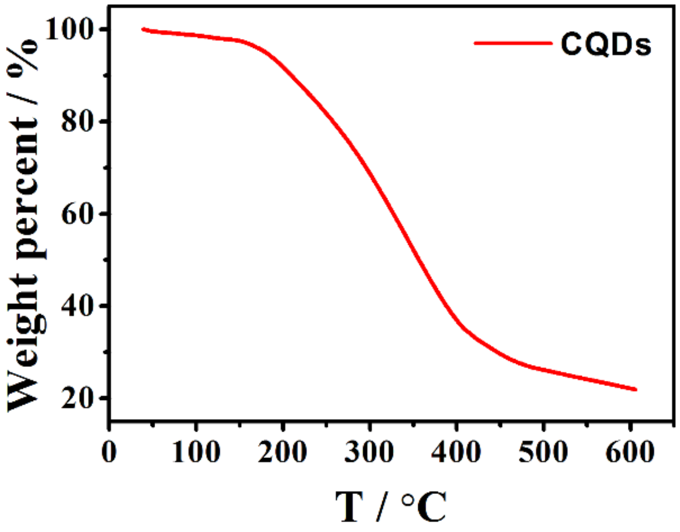

Figure S4. Thermogravimetric curve of CQDs.

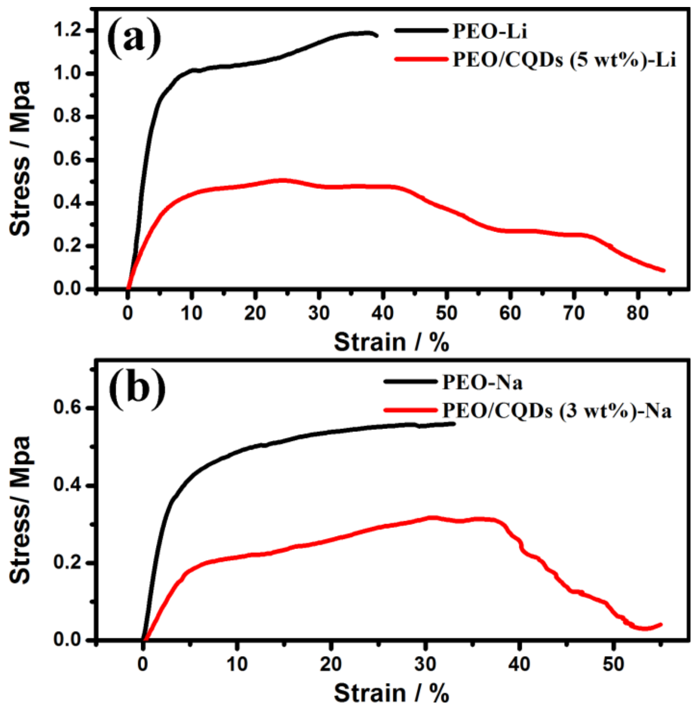

Figure S5. Stress-strain curves of PEO-Li and PEO/CQDs (5 wt%)-Li (a) and PEO-Na and PEO/CQDs (3 wt%)-Na (b).

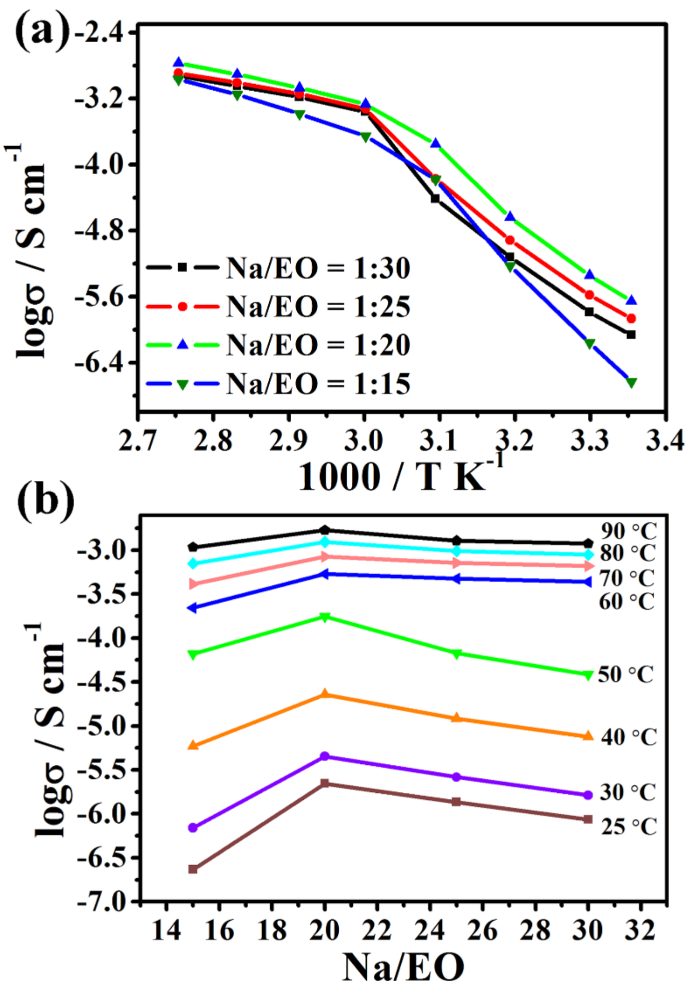

Figure S6. (a) Temperature dependent ionic conductivity for PEO-Na electrolytes. (b) Ionic conductivity for PEO-Na electrolyte with various $NaClO_4$ concentrations at different temperatures.

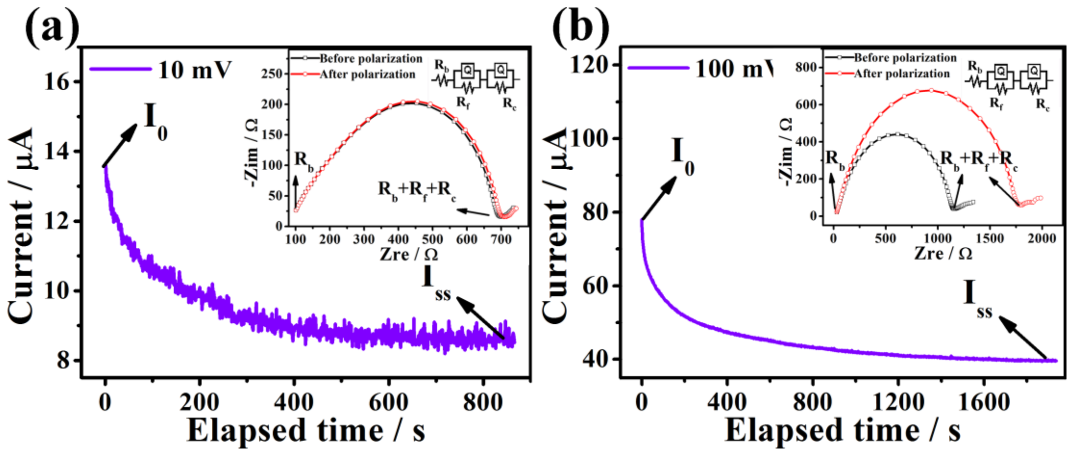

Figure S7. Chronoamperometry profiles and AC impedance spectra before and after polarization for (g) symmetric Li/Li cells with PEO-Li and (h) symmetric Na/Na cells with PEO-Na.

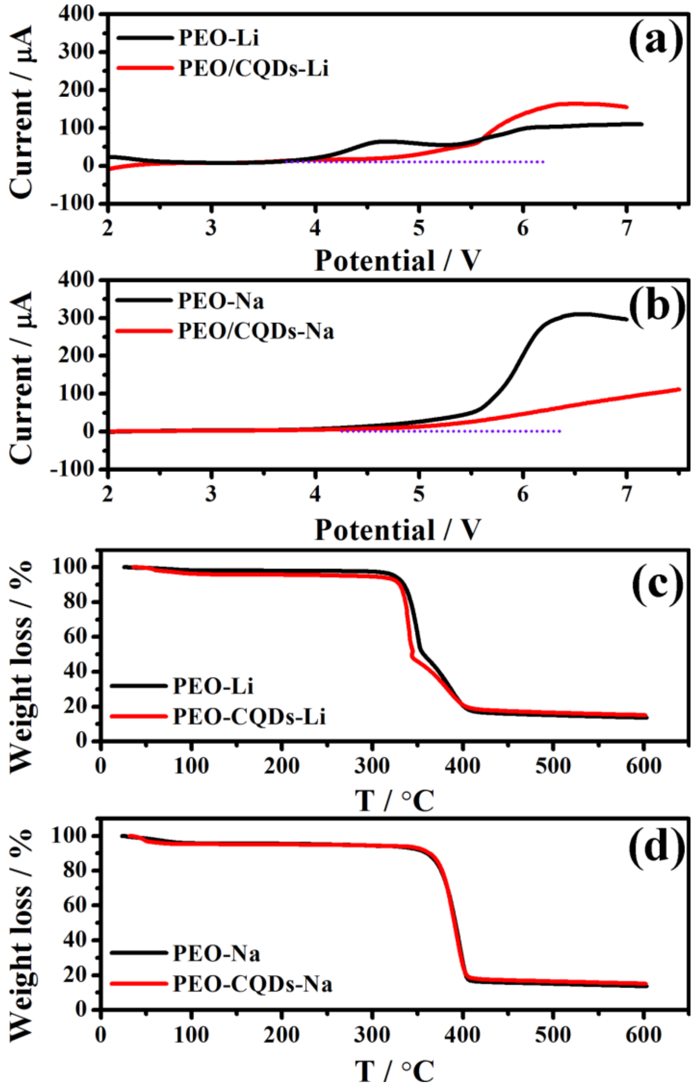

Figure S8. Electrochemical stability windows for (a) PEO-Li and PEO/CQDs-Li and (b) PEO-Na and PEO/CQDs-Na. Thermogravimetric curves for (c) PEO-Li and PEO/CQDs-Li and (d) PEO-Na and PEO/CQDs-Na.

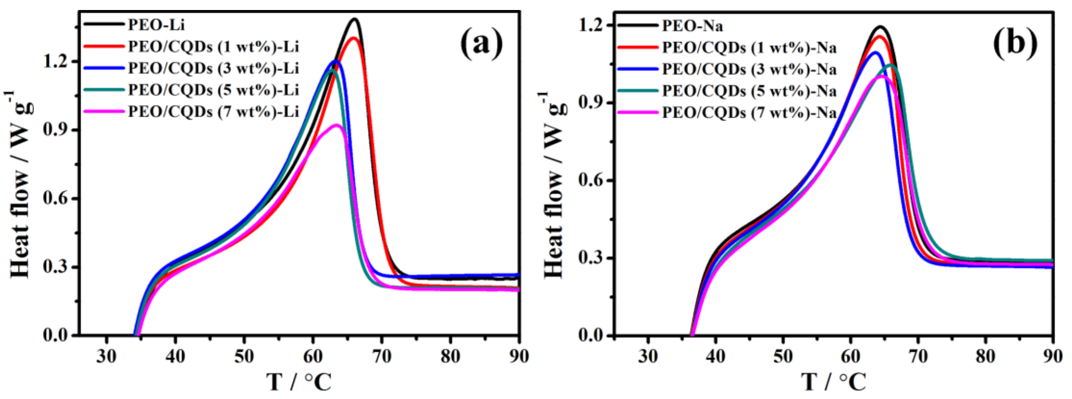

Figure S9. DSC curves for PEO/CQDs-Li (a) and PEO/CQDs-Na (b) with various CQDs contents.

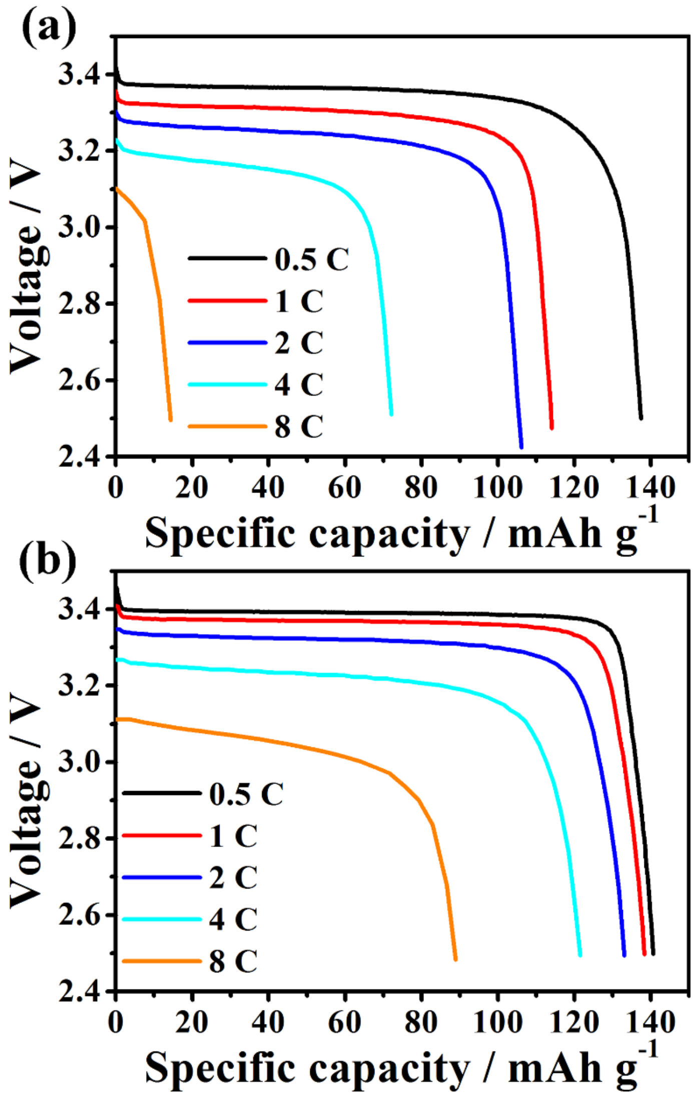

Figure S10. Discharge curves at various C-rates with a charge current density of 0.1 C for LFP/Li batteries using (a) PEO-Li electrolyte and (b) PEO/CQDs-Li electrolyte.

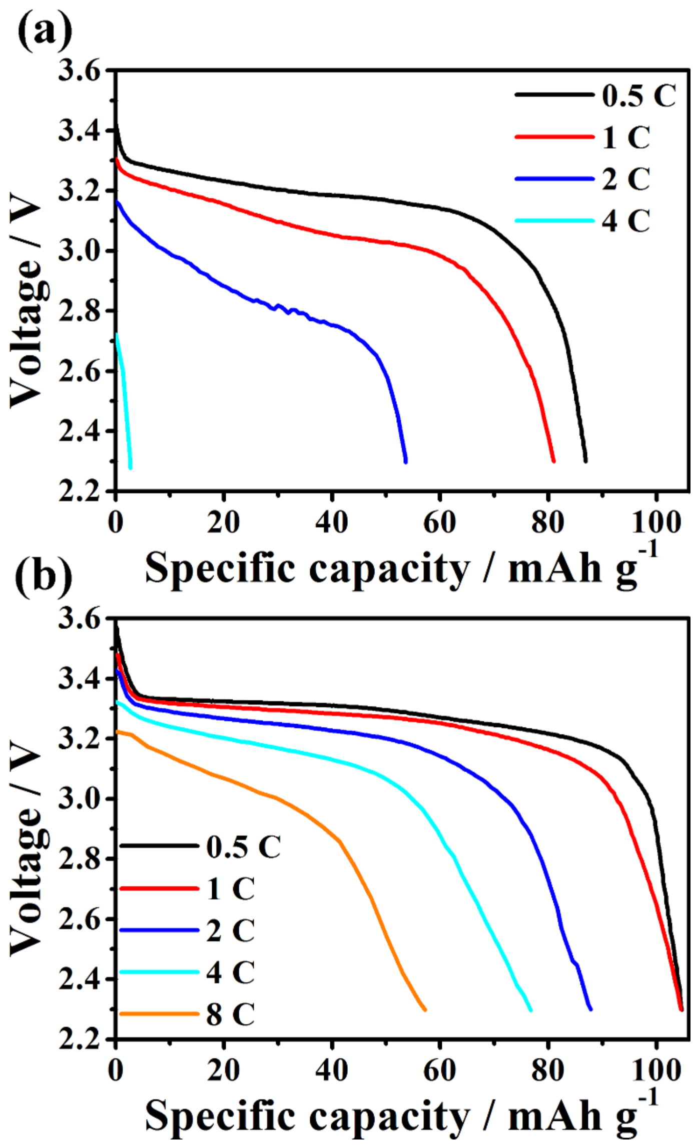

Figure S111. Discharge curves at various C-rates with a charge current density of 0.1 C for NVP/Li batteries using (a) PEO-Na electrolyte and (b) PEO/CQDs-Na electrolyte.

Table S1 Ionic conductivities for PEO/CQDs NPEs at different temperatures

T / ^{o}C	Ionic conductivities / S cm^{-1}			
	PEO-Li	PEO/CQDs-Li	PEO-Na	PEO/CQDs-Na
25	6.39×10^{-6}	1.39×10^{-4}	2.21×10^{-6}	7.17×10^{-5}
60	8.44×10^{-4}	2.57×10^{-3}	5.37×10^{-4}	1.28×10^{-3}
90	3.57×10^{-3}	9.50×10^{-3}	1.69×10^{-3}	2.82×10^{-3}

Table S2 Comparison of ion conductivity of PEO/CQDs NPEs with other nanofiller doped PEO polymer electrolytes

Ref.	Sample	Ionic conductivity / S cm^{-1}	Temperature / °C
This work	PEO/CQDs-Li	1.39×10^{-4}	25
[1]	PEO/SiO_2-Li	4.4×10^{-5}	30
[2]	PEO/$Li_{10}GeP_2S_{12}$	1.18×10^{-5}	25
[3]	PEO/clay-CNT-Li	2.07×10^{-5}	25
This work	PEO/CQDs-Na	7.17×10^{-5}	25
[4]	PEO/TiO2-Na	~2×10^{-5}	25
[5]	PEO/ZrO2-Na	6.96×10^{-5}	25
[6]	PEO/SiO2-Na	8.4×10^{-5}	25

[1] D. C. Lin, W. Liu, Y. Y. Liu, H. R. Lee, P. C. Hsu, K. Liu, Y. Cui, Nano letters 2015, 16, 459.

[2] Y. R. Zhao, C. Wu, G. Peng, X. T. Chen, X. Y. Yao, Y. Bai, F. Wu, S. J. Chen, X. X. Xu, J. Power Sources 2016, 301, 47.

[3] C. Y. Tang, K. Hackenberg, Q. Fu, P. M. Ajayan, H. Ardebili, Nano letters, 2012, 12, 1152.

[4] Y. L. Ni'mah, M. Y. Cheng, J. H. Cheng, J. Rick, B. J. Hwang, J. Power Sources, 2015, 278, 375.

[5] A. Dey, T. Ghoshal, S. Karan, S. K. De, Journal of Applied Physics, 2011, 110, 043707.

[6] S. F. Song, M. Kotobuki, F. Zheng, C. H. Xu, S. V. Savilov, N. Hu, L. Lu, Y. Wang, W. D. Z. Li, J. Mater. Chem. A, 2017, 5, 6424.

Table S3 Thermal data for PEO/CQDs-Li with various CQDs contents

Sample	T_m / ^{o}C	ΔH_m / J g^{-1}	χ_c
PEO-Li	66.03	132.92	68.16%
PEO/CQDs (1 wt%)-Li	65.95	123.39	63.28%
PEO/CQDs (3 wt%)-Li	63.28	109.31	56.06%
PEO/CQDs (5 wt%)-Li	62.80	107.83	55.30%
PEO/CQDs (7 wt%)-Li	63.45	96.78	49.63%

Table S4 Thermal data for PEO/CQDs-Na with various CQDs contents

Sample	T_m / ^{o}C	ΔH_m / J g^{-1}	χ_c
PEO-Na	64.38	106.86	54.80%
PEO/CQDs (1 wt%)-Na	64.29	104.43	53.55%
PEO/CQDs (3 wt%)-Na	63.66	98.22	50.36%
PEO/CQDs (5 wt%)-Na	65.91	98.17	50.34%
PEO/CQDs (7 wt%)-Na	64.65	95.98	49.22%

ΔH_m can be calculated from the integral area of each melting curve [7].

$\boldsymbol{\chi_c}$ is the ratio of the melting enthalpy of samples and 100% crystalline PEO (ΔH_m = 195 J g^{-1} given in Ref. [8]), taking into account the PEO volume fraction.

[7] Y. Tong, L. Chen, X. He, Y. Chen, *Electrochim. Acta* **2014,** 118, 33–40.

[8] L.O. Griffin, *Physical Constants of Linear Homopolymers*, Springer Verlag, Berlin, 1986.

Table S5 Free anion to ion pair ratio, indicating degree of dissociation of $LiClO_4$ and $NaClO_4$ in different polymer electrolytes

	PEO-Li	PEO/CQDs-Li	PEO-Na	PEO/CQDs-Na
Free anion	82.5%	92.7%	86.1%	97.9%
Ion pair	14.5%	7.3%	13.9%	2.1%
